# Upregulation of Scavenger Receptor BI by Hepatic Nuclear Factor 4**α** through a Peroxisome Proliferator-Activated Receptor **γ**-Dependent Mechanism in Liver

**DOI:** 10.1155/2011/164925

**Published:** 2011-11-14

**Authors:** Yi Zhang, Chen Shen, Ding Ai, Xuefen Xie, Yi Zhu

**Affiliations:** Department of Physiology and Pathophysiology, Peking University Health Science Center, Key Laboratory of Molecular Cardiovascular Sciences of Education Ministry, Beijing 100191, China

## Abstract

Hepatic nuclear factor 4**α** (HNF4**α**) modulates the transcriptional activation of numerous metabolic genes in liver. In this study, gene-array analysis revealed that HNF4**α** overexpression increased peroxisome proliferator-activated receptor**γ** (PPAR**γ**) greatly in cultured rat primary hepatocytes. PPAR-response-element-driven reporter gene expression could be elevated by HNF4**α**. Bioinformatics analysis revealed a high-affinity HNF4**α** binding site in the human PPAR**γ**2 promoter and *in vitro* experiments showed that this promoter could be transactivated by HNF4**α**. The presence of HNF4**α** on the promoter was then confirmed by ChIP assay. *In vivo*, hepatic overexpression of HNF4**α** decreased cholesterol levels both in plasma and liver and several hepatic genes related to cholesterol metabolism, including scavenger receptor BI (SR-BI), were upregulated. The upregulation of SR-BI by HNF4**α** could be inhibited by a PPAR**γ** antagonist *in vitro*. In conclusion, HNF4**α** regulates cholesterol metabolism in rat by modulating the expression of SR-BI in the liver, in which the upregulation of PPAR**γ** was involved.

## 1. Introduction

Nuclear receptors are ligand-activated transcription factors that regulate such diverse physiological processes as reproduction, development, and metabolism. Hepatic nuclear factor 4*α* (HNF4*α*) is a member of the nuclear receptor superfamily and plays an essential role in development and function in different organs, including liver, kidney, intestine, and pancreatic *β* cells. HNF4*α* contributes to gene regulation of the liver and pancreatic islet by binding directly to many actively transcribed genes [1]. The role of HNF4*α* as a metabolic transcriptional regulator in both the liver and pancreas has been uncovered by transcriptome analysis. Like other transcription factors, HNF4*α* modulates transcriptional activation of genes involved in transportation and metabolism of glucose, amino acids, lipids, and vitamins and in bile acid biosynthesis. HNF4*α* was once considered an orphan nuclear receptor because its endogenous ligand was not clear. Structural biology evidence suggests that HNF4*α* can bind to the C14 to C18 long-chain fatty acids [2, 3]. Recently, Sladek group reported that linoleic acid could bind with HNF4*α*, but ligand occupancy appeared not having a significant effect on its transcriptional activity [4]. Thus, HNF4*α* may be responsible for a change in metabolic status (e.g., changes in fatty acid levels) beyond direct transcriptional modulation. 

Clinical evidence revealed that loss-of-function mutations in HNF4*α* cause maturity onset diabetes of the young 1 (MODY1) [5], a type of early-onset diabetes with pancreatic *β* cell dysfunction. Patients with MODY1 have decreased blood triglycerides and cholesterol levels [6]. Gene variants of HNF4*α* are also associated with type 2 diabetes [7, 8]. The mechanism underlying hypolipidemia remains poorly understood. HNF4*α*-null mice die during embryogenesis [9], and mice lacking hepatic HNF4*α* expression showed increased lipid levels in the liver and greatly disturbed lipid metabolism. Liver-specific HNF4*α*-knockout mice showed profoundly decreased plasma levels of cholesterol, high-density lipoprotein-cholesterol (HDL-C), and triglycerides and elevated plasma level of bile acid [10]. Despite the investigation of numerous genes, the pattern of gene regulation of lipid homeostasis by overexpression of HNF4*α* is still unclear.

Previously, we reported that hyperinsulinemia downregulated HNF4*α* and its target genes through the upregulation of sterol responsive element binding proteins (SREBPs), other important transcriptional factors regulating cholesterol and fatty acid metabolism [11]. To further investigate the transcription profile of HNF4*α* in hepatocytes and its crosstalk with other transcription factors, we used a gain-of function model with adenovirus encoding HNF4*α* (Ad-HNF4*α*) and microarray analysis in hepatocytes and rat plasma and liver. Hepatic overexpression of HNF4*α* led to lower cholesterol levels both in plasma and liver, and another important metabolic nuclear receptor, peroxisome proliferator-activated receptor *γ* (PPAR*γ*), and a scavenger receptor SR-BI were involved in the process. Our findings suggest that hepatic HNF4*α* has a more potent role in cholesterol than glucose and triglycerides metabolism.

## 2. Material and Methods

### 2.1. Cell Culture and Reagents

The human hepatoma cell line HepG2 was maintained in Dulbecco's modified Eagle's medium (DMEM) containing 10% (vol/vol) fetal bovine serum (FBS, Hyclone, Logan, Utah, USA). HEK293 cells were cultured in DMEM with 5% FBS. Primary isolated and cultured hepatocytes were obtained from 8-week-old male Sprague-Dawley rats by the two-step collagenase perfusion technique [12]. Cells (3 × 10^6^) were plated on 60-mm rat tail collagen- (Millipore-) coated dishes containing medium 1640 supplemented with 20 mM HEPES, 1 mM sodium pyruvate, and 10% FBS for 3 days before treatment. The cells were infected with the adenovirus described below for 24 hr, then cultured in medium containing the PPAR*γ* antagonist BADGE (10 mM, Sigma, St. Louis, Mo, USA) or the same volume of relevant vehicle DMSO for an additional 24 hr. All cells were maintained at 37°C in 5% CO_2_, 95% air.

### 2.2. Adenovirus Construction and Infection

Ad-HNF4*α* was generated by use of the Adeno-X*™* Expression System 2 (Qbiogene, Carlsbad, Calif, USA). The adenovirus vector was based on replication-deficient E1 and E3 adenovirus under the transcriptional control of the cytomegalovirus promoter. The cDNA encoding a full-length rat HNF4*α* was obtained by digestion from the pcDNR-CMV-rHNF4*α* construct, then inserted into the pLP-Adeno-CMV construct under the control of the Cre/loxP system. DNA linearized by PacI digestion was packaged into the virus in HEK293 cells. The recombinant adenovirus (Ad-CMV-rHNF4*α*) was grown in HEK293 cells, purified, and titrated according to the manufacturer's instructions. HepG2 and rat primary hepatocytes were incubated with recombinant adenovirus at a multiplicity of infection (MOI) of 20 for 24 hr before experiments. 

### 2.3. RNA Extracts and Quantitative Real-Time PCR Analysis

Total RNA was extracted by the Trizol Reagent method (Applygen, Beijing, China). The isolated RNA was converted into cDNA. Quantitative RT-PCR with the Brilliant SYBR Green QPCR system involved use of *β*-actin as an internal control with the MX3000P QPCR detection system (Stratagene, La Jolla, Calif, USA). The primer sequences are in the supplementary tables. In a separate experiment, the extracted total RNA underwent PCR array of liver-related genes (SuperArray, SABiosciences, Frederick, Md, USA) by Kangcheng Inc. (Beijing, China).

### 2.4. Western Blot Analysis

Cell lysates and rat liver extracts were resolved by 10% SDS-PAGE and transferred to a nitrocellulose membrane. Protein expression of sEH, GAPDH and *β*-actin was detected by use of polyclonal antibodies anti-HNF4*α* (Santa Cruz Biotechnology, Santa Cruz, Calif, USA), anti-SR-BI (NOVUS Biologicals, Littleton, Colo, USA) and anti-*β*-actin (Bioss, Beijing, China) followed by horseradish peroxidase-conjugated secondary antibody. The protein bands were visualized by the ECL detection system (Amersham, Stockholm, Sweden), and the densities of the bands were quantified by computer-assisted image analysis (NIH Image J). 

### 2.5. Promoter Reporter Assay

A 1.6-kb fragment of PPAR*γ*2 promoter was cloned by PCR from the genome of the HEK293 cell line with the oligonucleotide primers 5′-TCCAGAAGTGAGACCCTTTG- AG-3′ and 5′-CATGGAATAGGGGTTTGCTGTAAT-3′. The amplified fragment was sequenced and then inserted into the EZ-T construct (Genestar, Beijing, China), then cloned into the reporter vector pGL3 (Promega, Madison, Wis, USA) on the *Sma*I and *Kpn*I sites and named PPAR*γ*2-(−1505)-Luc. PPAR*γ*2-(−893)-Luc and PPAR*γ*2-(−502)-Luc vectors were constructed by the same strategy. Transfection experiments were carried out in 24-well plates with use of JetPEITM (PolyPlus-transfection, Illkirch, France). Luciferase and *β*-galactosidase assays followed the manufacturer's instructions (Promega, Madison, Wis, USA).

### 2.6. Chromatin Immunoprecipitation (ChIP) Assay

ChIP assays were performed as described [13]. In brief, HepG2 cells were cross-linked and sonicated, then underwent immunoprecipitation (IP) with polyclonal anti-HNF4*α*. Normal IgG was used as an IP control, and the supernatant was an input control. After digestion with proteinase K, the resting DNA was extracted, and the PPAR*γ*2 promoter containing the HNF4*α* consensus element was amplified by PCR with the primers 5′-TGACAAGACCTGCTCC-3′ and 5′-TACGCTGTTAGGTTGG-3′. The resulting DNA was resolved on 2% agarose gel and stained with ethidium bromide. 

### 2.7. Animal Experiment

The investigation conformed to the Guide for the Care and Use of Laboratory Animals published by the US National Institutes of Health (NIH Publication No. 85–23, revised 1996). The animal experimental protocol was approved by the Peking University Institutional Animal Care and Use Committee. Sprague-Dawley rats were fed standard laboratory chow and tap water *ad libitum* and bred under a 12-h light/12-h dark cycle. The 8-week-old (~200 g) male rats were injected with Ad-rHNF4*α* at 1 × 10^9^ plaque-forming units in 0.5-mL saline through the tail vein. The same amount of Ad-GFP was injected in the control group (*n* = 6). Levels of plasma glucose were measured by use of a portable glucometer (ACCU-CHEK II; Roche Diagnostics, Basel, Swiss). Seven days after the injection, the rats fasted for 6 hr and then were anesthetized and killed. Blood samples were collected from the saphenous vein, and plasma levels of lipoproteins and insulin were measured. Liver tissues were dissected after a PBS rinse and stored at −80°C.

### 2.8. Measurement of Plasma and Tissue Lipids

Rat plasma samples were collected, and lipoproteins were separated by fast performance liquid chromatography (FPLC) (Pharmacia Biotech, Sweden). The levels of cholesterol and triglycerides in plasma or the FPLC fractions were detected by use of an automated clinical chemistry analyzer kit (Biosino Biotech Inc., Beijing, China). For quantification of liver cholesterol and triglycerides, approximately 100 mg liver was homogenized and the extraction was dissolved with chloroform : methanol (2 : 1). The lipid fractions were dried under nitrogen gas and re-solubilized by phosphate buffered saline containing 1% Triton X-100 before measurement of cholesterol and triglyceride levels.

### 2.9. Statistical Analysis

Data were analyzed by the unpaired Student *t* test, one-way ANOVA, or Mann-Whitney test (GraphPad Prism4 software). All values are expressed as mean ± SEM. Differences were considered statistically significant at *P* < 0.05. 

## 3. Results

### 3.1. Hepatic HNF4*α* Induces PPAR*γ* Upregulation and Activation

**Figure 1:**
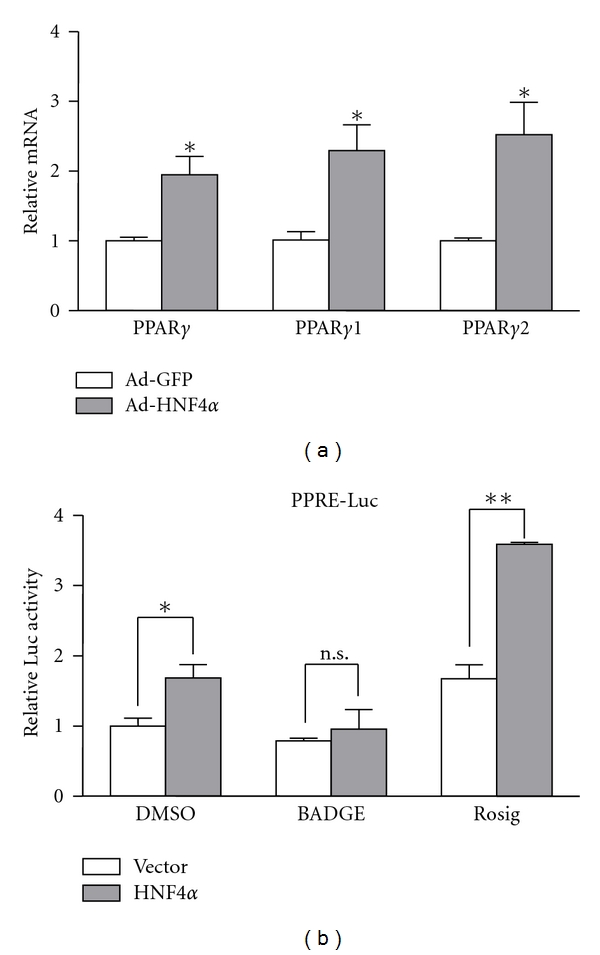
Hepatic nuclear factor 4*α*(HNF4*α*) induced peroxisome proliferator-activated receptor *γ* (PPAR*γ*) transcription activity in hepatocytes. (a) Quantitative RT-PCR (qRT-PCR) analysis of mRNA levels of PPAR*γ* and its subtypes PPAR*γ*1 and PPAR*γ*2 in isolated primary hepatocytes. (b) HepG2 cells were cotransfected with plasmids of 3 × PPRE-Luc reporter plasmid with pMT7-HNF4*α* or vehicle plasmid for 24 hr, and luciferase activity with PPAR*γ* antagonist BADGE and agonist rosiglitazone (Rosig) was measured. The *β*-gal plasmid was cotransfected as a transfection control. Promoter activities were measured by relative luciferase activity, which was normalized to that of *β*-gal. Results are mean ± SEM mRNA levels normalized to that of *β*-actin and expressed as fold of control group (DMSO). Results are representative of 3 independent experiments. *P < 0.05, **P < 0.01.

Previously, we reported that hepatic HNF4*α* was downregulated in db/db diabetic mice [14]. To further investigate the role of HNF4*α* in liver metabolism, we overexpressed HNF4*α* in rat primary hepatocytes by Ad-rHNF4*α* infection and analyzed the expression of liver-related genes by PCR array. As shown in Table 1, known HNF4*α* target genes, such as glucose-6-phosphatase, catalytic subunit (G6pc) and liver type of pyruvate kinase (L-PK), were upregulated by HNF4*α* overexpression, as reported previously [14]. Surprisingly, we found a 19.29-fold increase of PPAR*γ* expression with Ad-rHNF4*α* infection relative to the Ad-GFP control (Table 1). Meanwhile, the expression of Srebf1, a gene encoding a major transcriptional regulator SREBP-1c, which is important in triglyceride metabolism, showed a 1.53-fold decrease in expression (data not shown). The upregulation of PPAR*γ*, including PPAR*γ*1 and PPAR*γ*2, by HNF4*α* overexpression was confirmed by quantitative real-time PCR (Figure 1(a)). To assess whether HNF-4*α* contributes to the transactivation of PPAR*γ*, we performed PPRE-driven luciferase reporter assays in the human hepatic cell line HepG2. Rosiglitazone, the PPAR*γ* agonist, was used as a positive control. Compared with control cells cotransfected with vehicle plasmid, the PPRE-luc activity was induced by HNF-4*α* co-transfection, and BADGE, the PPAR*γ* antagonist, could diminish this effect (Figure 1(b)), which suggests that HNF-4*α* transactivated PPRE-luc activity through PPAR*γ*. 

### 3.2. HNF4*α* Is Involved in PPAR*γ*2 Transactivation

**Figure 2:**
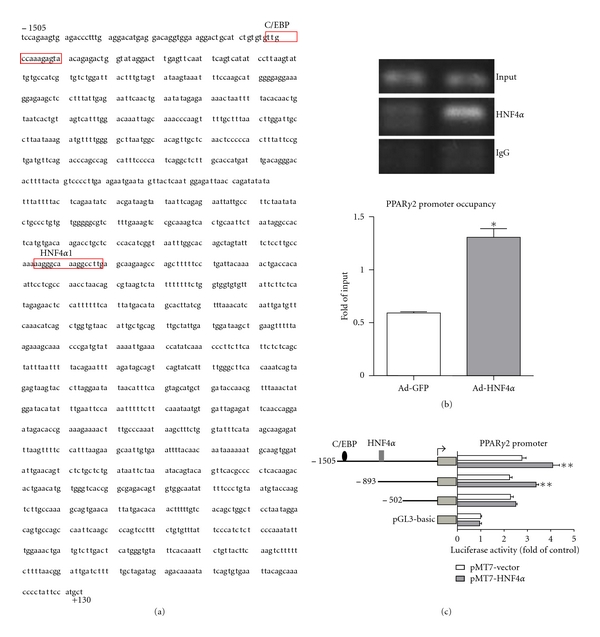
HNF4*α* transactivated PPAR*γ*2 promoter in hepatocytes. (a) Sequence of the 1.6-kb cloned human PPAR*γ*2 promoter and sketch of putative HNF4*α* binding site. (b) HepG2 cells were infected with or without adenovirus HNF4*α* (Ad-HNF4*α*) for 24 hr and underwent chromatin immunopreciptitation (ChIP) assay with anti-HNF4*α* antibody; normal rabbit IgG was used in control experiments. qPCR was used with PPAR*γ* promoter-specific primers to detect binding of HNF4*α* to the PPAR*γ*2 promoter. The DNA levels were normalized to that of input and expressed as fold of the control group. *P < 0.05. (c) HepG2 cells were cotransfected with plasmids of PPAR*γ*2p1505-Luc, PPAR*γ*2p893-Luc, or PPAR*γ*2p502-Luc with pMT7-HNF4*α* or vehicle plasmid. The *β*-gal plasmid was cotransfected as a transfection control. Promoter activities were measured by relative luciferase activity, with the level normalized to that of *β*-gal, from 3 independent experiments, each performed in triplicate. Data are mean ± SD. **P < 0.01 compared with control.

Sequence comparison by BLAST analysis revealed more than 80% similarity in the PPAR*γ*2 promoter but not PPAR*γ*1 promoter between human and rat. We identified a putative HNF4*α* site on the human PPAR*γ*2 promoter (in the region of −842 to −827) by the prediction program of the CREAD platform (PKUHSC) (Figure 2(a)). ChIP assay revealed that HNF4*α* could bind to the predicted HNF4*α* binding site on the PPAR*γ*2 promoter (Figure 2(b)). Moreover, quantitative PCR with PPAR*γ*2 promoter sequence-specific primers revealed that HNF4*α* overexpression enhanced the promoter occupancy of HNF4*α* on the PPAR*γ*2 promoter (Figure 2(b)). Transient transfection assay with a series of 5′-deletion reporter constructs containing different lengths of the PPAR*γ*2 promoter revealed that deletion of 893 to 502 bp in the promoter region led to a large decrease in the ability of HNF4*α* to transactivate the PPAR*γ*2 promoter (Figure 2(c)).

### 3.3. Hepatic Overexpression of HNF4*α* in Rat Lowered Plasma and Liver Cholesterol Levels

**Figure 3:**
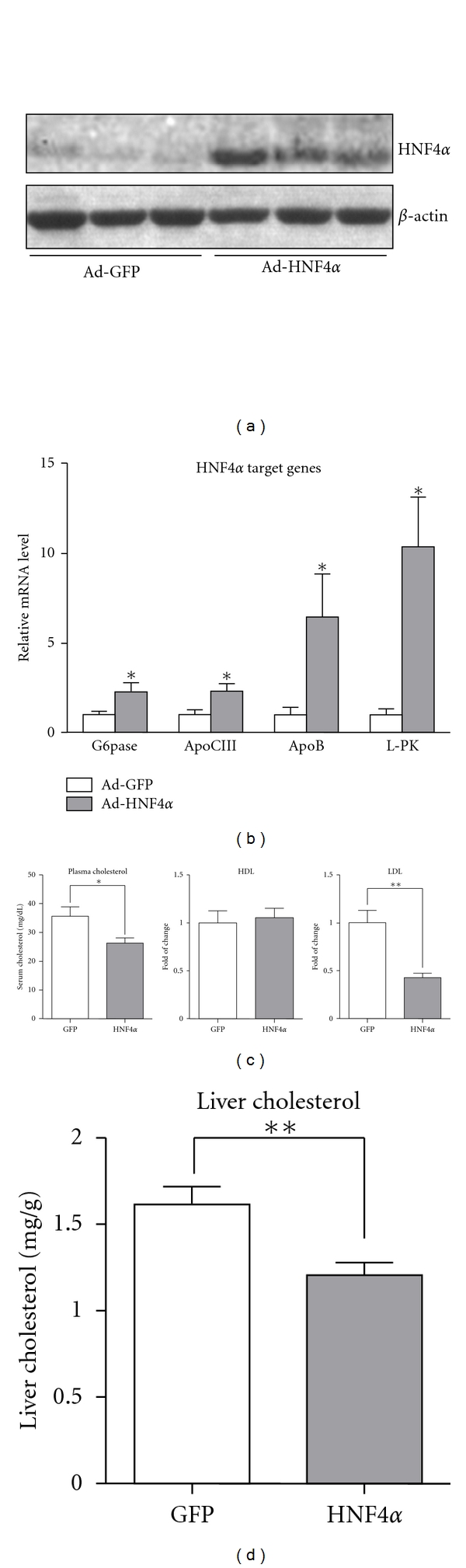
Hepatic overexpression of HNF4*α* in rats decreased cholesterol levels in both plasma and liver. Rats were intravenously injected with Ad-HNF4*α* or Ad-GFP (*n* = 6), then killed 7 days post-infection. (a) Liver extracts were used to determine protein expression by western blot analysis with antibodies against HNF4*α* or *β*-actin. (b) Relative mRNA levels of hepatic HNF4*α* target genes, including G6p, Apoc3, Apob, and L-pk, were examined by qPCR. (c) Plasma cholesterol, high-density lipoprotein cholesterol (HDL-C), and low-density lipoprotein cholesterol (LDL-C) levels were determined. (d) Liver cholesterol was measured in lipid content of rat liver. Data are mean ± SEM, *P < 0.05, **P < 0.01 versus Ad-GFP infection.

To ascertain the role of HNF4*α*-upregulated PPAR*γ* in liver *in vivo*, rats were injected with Ad-HNF4*α* or Ad-GFP intravenously and killed 7 days later. Overexpression of HNF4*α* in liver significantly increased hepatic HNF4*α* protein level (Figure 3(a)) as compared with Ad-GFP-infected controls. mRNA levels of HNF4*α* target genes (Pepck, G6p, Apob, Apoc3, and L-pk) in liver were elevated by Ad-HNF4*α* (Figure 3(b)). The rats showed normal body weight, liver weight, plasma insulin level (Table 2), and oral glucose tolerance (data not shown). Plasma level of triglycerides was increased but not significantly (Table 2). Oil-red O staining showed no lipid accumulation in the liver (data not shown). However, cholesterol levels were reduced in both plasma and liver (Figures 3(c) and 3(d)). Plasma lipoprotein analysis revealed decreased LDL-C but not HDL-C fraction (Figure 3(c)).

### 3.4. Selective Regulation of Hepatic Cholesterol-Regulatory Genes by HNF4*α* in Liver

**Figure 4:**
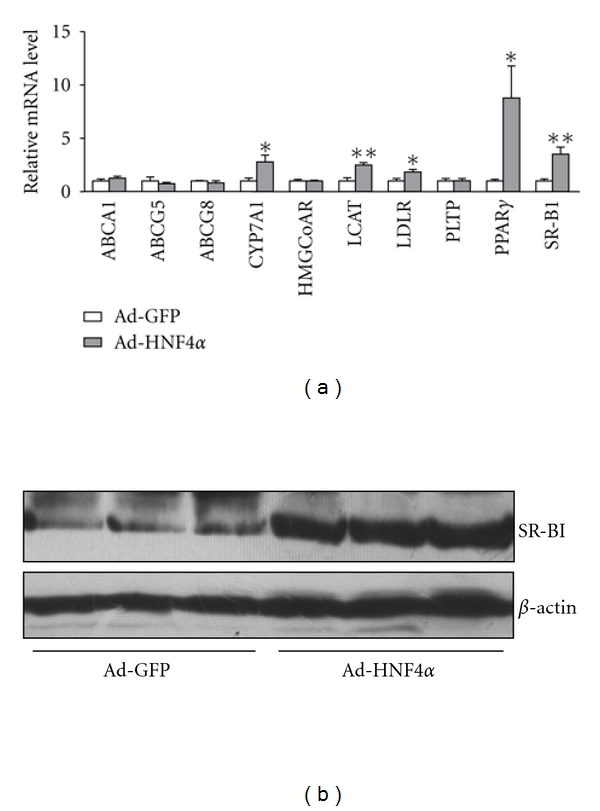
Expression pattern of hepatic cholesterol-regulatory genes under HNF4*α* overexpression in rat liver. (a) Total RNA was extracted from rat liver and the mRNA levels of hepatic cholesterol-regulatory genes, including HMGCoAR, LCAT, PLTP, LDLR, SR-BI, ABCA1, ABCG5, ABCG8, and CYP7A1, were measured by qPCR. Data are mean ± SEM mRNA levels normalized to that of *β*-actin and expressed as fold of GFP-infected group. *P < 0.05. (b) Rat liver extracts were examined by western blot analysis with antibodies against SR-BI or *β*-actin.

To study the mechanism of HNF4*α*-regulated cholesterol metabolism in liver, we examined the expression of pivotal genes governing liver cholesterol metabolism in rat liver with HNF4*α* overexpression. qPCR results revealed elevated mRNA levels of hepatic PPAR*γ* and its known target gene CYP7*α*1 [15], as expected (Figure 4(a)). Genes regulating lipoprotein uptake, such as LDL receptor and scavenger receptor class B type I (SR-BI), were also upregulated. However, genes regulating cholesterol *de novo* synthesis, efflux and metabolism, such as ABCA1, ABCG5, ABCG8, and HMG-CoA reductase showed no regulation by HNF4*α*. Intriguingly, the mRNA level of lecithin:cholesterol acyltransferase (LCAT), which converts cholesterol and phosphatidylcholines (lecithins) to cholesteryl esters and lysophosphatidylcholines on the surface of high-density lipoproteins [16], was elevated by HNF4*α* overexpression (Figure 4(a)). Therefore, cholesterol uptake genes seemed to be regulated by HNF4*α* in liver, possibly via PPAR*γ* upregulation.

### 3.5. Upregulation of SR-BI by HNF4*α* Depended on PPAR*γ*

**Figure 5:**
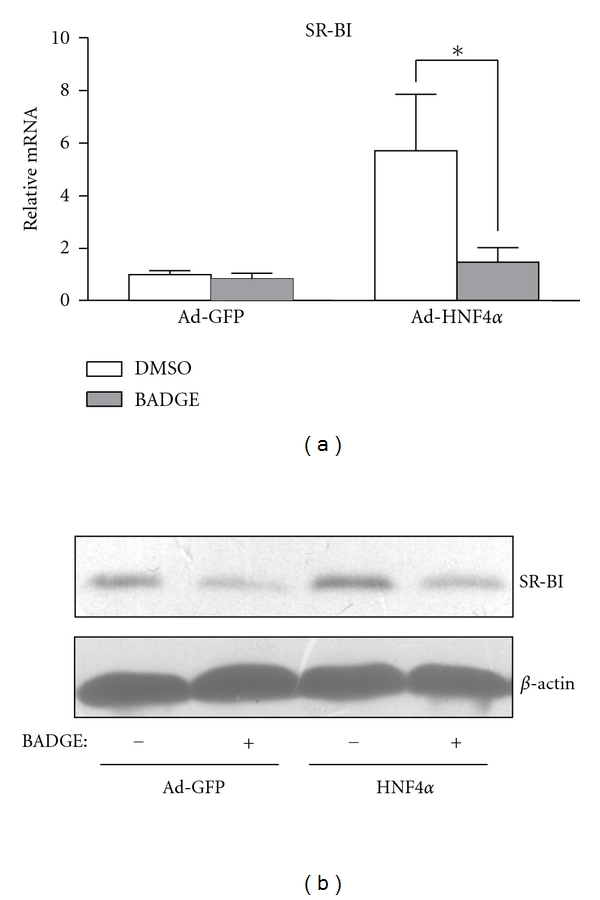
HNF4*α*-upregulated SR-BI depends on PPAR*γ*. Rat primary hepatocytes were pretreated with or without PPAR*γ* antagonist BADGE (10 *μ*M) for 30 min and then infected with Ad-HNF4*α* or Ad-GFP. (a) Real-time PCR analysis of mRNA expression and (b) western blot analysis of protein expression from 3 independent experiments. *β*-actin was used as an internal control. Data are mean ± SEM mRNA levels normalized to that of *β*-actin and expressed as fold of control group. *P < 0.05, **P < 0.01.

SR-BI was found regulated by activators of PPAR*γ* in human hepatocytes [17] and a PPRE was proved to exist in the human SR-BI promoter [18]. Western blot analysis confirmed the upregulation of SR-BI with HNF4*α* overexpression at the protein level *in vivo* in rat liver (Figure 4(b)) and *in vitro* in rat hepatocytes (Figure 5(b)). We further investigated the role of HNF4*α* in SR-BI upregulation in cultured hepatocytes. The PPAR*γ* antagonist BADGE could attenuate HNF4*α*-induced SR-BI expression at both mRNA and protein levels (Figures 5(a) and 5(b)), which suggests that HNF4*α* upregulates SR-BI in a PPAR*γ*-dependent manner.

## 4. Discussion

PPAR*γ* is a ligand-activated transcription factor that regulates diverse biological activities and plays major roles in many diseases, including diabetes mellitus, metabolic syndrome, and atherosclerosis [19]. It is highly expressed in adipose tissue, where it plays an essential role in fat storage and the differentiation of adipocytes [20]. However, PPAR*γ* is expressed at low levels in other tissues, including liver. An antidiabetic drug, pioglitazone, a known PPAR*γ* activator, significantly improved lipid metabolism and insulin responsiveness and reduced the hepatic inflammatory response [21]. Among the hepatic expression PPAR*γ* isoforms, though PPAR*γ*2 has lower expression amount than PPAR*γ*1, it is the only PPAR*γ* isoform which could be regulated at the transcriptional level by nutrition [22]. Furthermore, PPAR*γ*2 is the liver isoform that is ectopically induced in response to excess nutrition or genetic obesity [23]. Here, we show that PPAR*γ* is a direct target gene of HNF4*α* in rat, and HNF4*α* overexpression may achieve its cholesterol-lowering effect via PPAR*γ*-SR-BI upregulation. Martinez-Jimenez et al. showed that HNF4*α* can bind to and activate the PPAR*γ*1 promoter, and the transcriptional cofactor Hes6 interacted with HNF4*α* to prevent the hepatic transactivation of PPAR*γ*1 [24]. In addition to Martinez's work, our data demonstrate a functional HNF4-responsive element on the PPAR*γ*2 promoter. HNF4*α* could upregulate both PPAR*γ*1 and PPAR*γ*2 in our gain-of-function model with overexpression of HNF4*α*. We did not investigate the involvement of hepatic Hes6. Recently, HNF4*α* was proved to have the ability to bind with RXR*α*, a classical PPAR*γ* transcriptional cofactor [25]. The fold activation of PPAR*γ* by HNF4*α* overexpression differed *in vivo *and* in vitro *and that might result from the varied combinatiorial regulation of various factors including ligands and other transcription factors. The authors do not exclude the possibility that overexpression of HNF4*α* may change the expression pattern of PPAR*γ* transcription variants.

Expression of PPAR*γ* in the liver was augmented in murine steatosis, and adenovirus-mediated overexpression of PPAR*γ* in the liver provokes steatosis [26, 27]. Surprisingly, we did not observe any significant change in hepatic steatosis in Ad-HNF4*α*-treated rats. In a previous study, coexpression of HNF4*α* increased the promoter activity of PPAR*α* [28]. TZD18, a potent agonist with dual PPAR*α*/*γ* agonist activities, affected lipid homeostasis, thus leading to an antiatherogenic plasma lipid profile [29]. Our results showed reduced LDL-C level and upregulation of PPAR*γ* by HNF4*α*. HNF4*α* might be a dual PPAR*α*/*γ* activator, and overexpression of HNF4*α* may be effective in treatment of type 2 diabetes and dyslipidemia and in preventing atherosclerotic cardiovascular disease. 

Cholesterol homeostasis depends on proper control of cholesterol uptake, *de novo* synthesis, efflux, and metabolism. HNF4*α* is highly expressed in the liver and regulates numerous genes involved in energy metabolism. Decreased cholesterol, HDL cholesterol, and triglyceride levels found in the plasma of mice lacking hepatic HNF4*α* [10] suggests that HNF4*α* plays an important role in controlling hepatic lipid metabolism and transport. In our previous study, we found that HNF4*α* was sensitive to plasma insulin level and was downregulated by SREBPs in hyperinsulinemia diabetes [11] and hepatic SREBPs were downregulated by HNF4*α* overexpression in mice (unpublished data). Compared with knowledge from loss-of-function study [10], the one of the role HNF4*α* in gain-of-function animal models is limited. In the loss-of-function model of liver-specific HNF4*α* deficiency, authors attributed the decrease of the serum cholesterol to the reduced *de novo* cholesterol biosynthesis, VLDL secretion, and HDL biogenesis [10, 30]. However, our data revealed that hepatic HNF4*α* overexpression mainly affect the uptake of circulating cholesterol in rat liver. Overexpression of HNF4*α* in the liver lowered the plasma level of LDL-C but had little effect on HDL-C and triglyceride levels in plasma and liver. These data indicate that HNF4*α* overexpression might have a moderate cholesterol-lowering effect. Further dissection of genes pivotal in the control of hepatic cholesterol homeostasis in the rat liver revealed that HNF4*α* overexpression mainly enhanced the expression of genes involved in cholesterol uptake. This result is in accordance with those of a recent study of a similar gain-of-function mouse model [30]. 

The role of SR-BI in the hepatic hypocholesterolemic effect under hepatic HNF4*α* deficiency is still controversial: Hayhurst et al. attributed the cholesterol-lowering effect to increased hepatic SR-BI expression [10] while in an acute liver-specific loss of function model, Yin et al. demonstrated an elevation in liver SR-BI level [30]. In compensated for the mice data, we have provided *in vivo* and *in vitro* gain-of-function data on rats.

We found the mRNA level of LDLR and SR-BI but not ABCG5 were upregulated. Moreover, the fold upregulation of SR-BI is higher than LDLR in our *in vivo* data. It has been reported previously that HNF4*α* could interact with SREBP-2 on the sterol-response-element (SRE) of the LDLR promoter and hence modulate its transcriptional activity [28]. The hepatic upregulation of SR-BI was found to decrease LDL-C and HDL-C content in mice [31]. We further found overexpression of HNF4*α* upregulated the protein level of SR-BI *in vivo*. Our finding together with the data from the HNF4*α*-knockout mice research suggests that the regulation of SR-BI by HNF4*α* might be indirect. In this study, we showed that overexpression of HNF4*α* in isolated rat primary hepatocytes induced SR-BI, whereas inhibition of PPAR*γ* diminished this effect, which suggests the involvement of HNF4*α*-induced PPAR*γ* transactivation. In addition, rosiglitazone, a known PPAR*γ*-agonist, significantly augmented the stimulation effect of HNF4*α* on SR-BI mRNA level. The synergistic effect of PPAR*γ*-ligand and HNF4*α* overexpression gave us further evidence to support the idea that the elevation of SR-BI induced by enhanced HNF4*α* expression level is due to the HNF4*α*-induced transactivation of PPAR*γ*. These findings indicate that HNF4*α* induced upregulation with the involvement of PPAR*γ* transactivation in liver might, at least in part, account for the cholesterol-lowering effect of hepatic HNF4*α* overexpression. 

We noticed that there was a significant difference of basal glucose levels with hepatic HNF4*α* overexpression than the control rats. This might attribute to the elevation of important glucose metabolic genes, including *Pepck, G6pc*, and *L-pk*. They are known HNF4*α*-target genes and can be upregulated to maintain an appropriate circulating glucose level while fasting. However, we did not observe any interruption caused by hepatic HNF4*α* overexpression in the oral glucose tolerance test on rats. The role of hepatic HNF4*α* in glucose metabolism is worth future investigation. 

Rat livers exposed to Ad-PPAR*γ* showed significantly less fibrosis than did controls [32], and overexpression of HNF4*α* alleviated hepatic fibrosis in rats with bile duct ligation [33]. HNF4*α* may prevent hepatic stellate cell activation and thus ameliorate liver steatosis through transactivation of PPAR*γ*. Because of evidence of the interaction between PPAR*γ*2 and HNF4*α* contributing to variation in insulin sensitivity in Mexican Americans [34], more work is needed to uncover the role of HNF4*α* and PPAR*γ* in various human diseases.

In summary, we demonstrate that HNF4*α* transcriptionally upregulates PPAR*γ*2 by directly binding to the PPAR*γ*2 promoter. We also reveal the regulatory role of HNF4*α* in liver lipid metabolism by modulating the expression of cholesterol metabolism-associated genes. These effects were, at least in part, due to the upregulation of SR-BI through the activation of PPAR*γ* by HNF4*α*.

## Figures and Tables

**Table 1:** Genes differentially regulated in rat primary hepatocytes by adenovirus HNF4*α* (Ad-HNF4*α*) relative to Ad-GFP infection according to the superarray analysis.

Accession No.	Name	Description	Fold Difference
NM_022180	HNF4*α*	Hepatocyte nuclear factor 4, alpha	333.14
NM_022215	GPD1	Glycerol-3-phosphate dehydrogenase 1 (soluble)	97.01
NM_013124	PPAR*γ*	Peroxisome proliferator activated receptor, gamma	19.29
NM_022852	PDX1	Pancreatic and duodenal homeobox gene 1	7.73
NM_013098	G6PC	Glucose-6-phosphatase, catalytic	5.54
NM_133380	IL4R	Interleukin 4 receptor	3.10
NM_012524	CEBP*α*	CCAAT/enhancer binding protein (C/EBP), alpha	3.05
NM_013091	TNFRSF1*α*	Tumor necrosis factor receptor superfamily, member 1a	2.23
NM_012565	GCK	Glucokinase	2.22
NM_012580	HMOX1	Heme oxygenase (decycling) 1	2.19
NM_138880	IFN*γ*	Interferon gamma	−2.01
NM_022944	INPPL1	Inositol polyphosphate phosphatase-like 1	−2.07
NM_022611	IL12*β*	Interleukin 12b	−2.17
NM_012544	ACE	Angiotensin 1 converting enzyme	−2.36
NM_031116	CCL5	Chemokine (C-C motif) ligand 5	−2.57
NM_012589	IL6	Interleukin 6	−4.47

**Table 2:** Body weight, organ weights, and plasma metabolic values in adenovirus-infected rats.

	Ad-GFP	Ad-HNF4*α*
Body weight (g)	204.1 ± 5.910	214.3 ± 5.877
Liver weight (g)	9.187 ± 0.5127	10.51 ± 0.5530
Liver weight to body weight	0.04493 ± 0.001696	0.04901 ± 0.002013
Insulin (uIU)	22.21 ± 0.7384	20.15 ± 1.647
Plasma triglycerides (mg/dl)	36.02 ± 4.798	46.94 ± 6.107
Liver triglycerides (mg/g·dl)	1.532 ± 0.3019	1.088 ± 0.0799
Basal glucose (mmol/dl)	5.167 ± 0.1626*	6.150 ± 0.1565

* *P* < 0.05 compared with control group.

## Abbreviations

ABC: ATP-binding cassette transporter
Apob: Apolipoprotein B;
Apoc3: Apolipoprotein C-III;
G6p: Glucose-6-phosphatase;
Hes6: Hairy enhancer of split 6;
HNF4*α*: Hepatocyte nuclear factor 4*α*;
LCAT: Lecithin:cholesterol acyltransferase;
L-pk: L-pyruvate kinase;
Pepck: Phosphoenolpyruvate carboxykinase;
PLTP: Phospholipid transfer protein
PPAR: Peroxisome proliferator activated receptor;
SR-BI: Scanvege receptor B type I
Srebf1: Sterol regulatory element binding factor-1.
